# Dr. McCormack’s Lecture

**Published:** 1906-01

**Authors:** 


Dr. McCormack's Lecture.—[By courtesy of the *State Journal of Medicine* we are permitted to see advance sheets of Dr. J. N. McCormack's address on "Organization," which will appear in the January number of that journal. We regret that we have not room to reproduce the address, but we clip the following from it:]

A county society is the only influence that can elevate the county profession in learning, brotherly love and helpfulness. The business side should receive careful attention. Let me advise you to begin your night fees at 8 p. m. that you may have time for study and recreation. Nine out of ten night calls are unnecessary. Patients can be educated to understand that they do not get first-class service from overworked and sleepy doctors.

The character of local organization determines all results. I will describe to you a typical county society meeting. When the time arrives most of the members are conspicuous by their absence. It reminds one of an old-fashioned Presbyterian prayer meeting, composed of a few good old fellows, who would have been good had they stayed at home. The president announces the program. You can usually count on three papers, one on typhoid fever, one on puerperal convulsions, and, at the beginning of the warm season, one on the diarrheal diseases of infancy. The first paper is read. It is usually a text-book article, and from an old text-book that with the reader should have passed through several revisions. The president then says: "Gentlemen, you have heard this very valuable paper. Who will open the discussion? Come, time is limited. Dr. Smith, will you open the discussion?" Dr. Smith slowly rises, and says: "I have not been attending the meetings as regularly as I should. I feel when I hear a paper like this I will never miss another. The paper I consider a very valuable contribution to medical literature. The author has covered the ground so fully that there hardly remains anything for me to say," and sits down. They go through such a farce as this, and call it a medical society. No wonder doctors do not come in. Who would?

Beside the great scientific and business problems there are minor conditions demanding attention in each community. The modern drug store is little more than a station for the dispensing of patent medicines and nostrums. The druggists need your counsel. They should be invited to your meetings, and together talk over the evil of counter-prescribing, refilling and substitution. At a recent meeting some one doubted that there was much substitution. I asked him to write six simple prescriptions for a glycero-phosphate and a messenger was sent to six different drug stores. Before the meeting adjourned, he returned with three filled correctly, two with cheap substitutes, and one consisting of commercial glycerin without a trace of medicinal agent. Require your druggists to get in line or stop patronizing them. As a final resort, doctors have found it necessary to dispense their own drugs in various parts of the country.

The clergymen should be invited to your meetings, and be made to appreciate your aims and co-operate with you in your struggle against nostrums and quacks. Holmes long ago said: "Quackery hobbles on two crutches, the tattle of old women and the testimonials of clergymen." The old women are not so much in evidence, but I hold in my hand the testimonials of clergymen attesting the value of Peruna. My son recently visited a girl sick in a fashionable female seminary, and found three girls taking Peruna. One had six bottles in the closet. When asked how she came to take it, she said: "O, my father uses it; he is a preacher, almost a doctor, you know. Why, he says he can take a little before he goes into the pulpit and he can preach much better—flies all over him." A physician recently told me he was called to see a prominent Ohio divine. After an examination the physician called his son and told him privately that his father was a confirmed alcoholic. "Impossible," cried the son, "my father is not only a minister, but a teetotaler and a temperance lecturer." "Can't help it," the doctor said, "he is drunk now. He has a diseased stomach and an enlarged liver from chronic alcoholism. Is there nothing your father takes?" "Absolutely nothing," the son said, "except a bottle or so of Peruna every day."

Let me also say that the time has come when ministers should no longer be treated like paupers. Take them off your free list. They are recipients of regular salaries, like your other patrons. The only reason for continuing gratuitous service on the part of the doctors is to bait the cow to catch the calf. I believe the ministry itself will welcome the independence.

In conclusion, let me say that I believe the doctor, as a representative of a great charitable and learned profession, should be the cleanest man in the community—clean in speech, clean in body, and clean in morals. He should be the best dressed man, as becomes his social standing. He should be the broadest and most cultured man in literature and science. He should be public-spirited—a model citizen. He should take this vow: "So long as God shall let me live I will never say an unkind word of a fellow-doctor." If a doctor may commit an unpardonable sin, it is to mistreat a young practitioner, and it is but a little milder sin to mistreat an old one.

